# Disability inclusion in African health systems’ responses during COVID-19: A scoping review

**DOI:** 10.4102/ajod.v12i0.1284

**Published:** 2023-12-21

**Authors:** Madri Engelbrecht, Yandisa Ngqangashe, Luphiwo Mduzana, Kate Sherry, Lieketseng Ned

**Affiliations:** 1 Centre for Disability and Rehabilitation Studies, Faculty of Medicine and Health Sciences, Stellenbosch University, Cape Town, South Africa; 2 BHSC Medical Orthotics and Prosthetics, Faculty of Health Sciences, Walter Sisulu University, Mthatha, South Africa

**Keywords:** disability inclusion, health systems’ responses, COVID-19, Africa, scoping review, deductive thematic analysis, healthcare access

## Abstract

**Background:**

People with disabilities often experience poorer access to healthcare because of multiple barriers even in non-crisis times, especially more so in low- and middle-income countries. The coronavirus disease 2019 (COVID-19) pandemic significantly constrained health systems, thus exacerbating access barriers. African health system responses to, and considerations made for people with disabilities during the pandemic have not been adequately examined to inform future inclusive practices during emergent and non-emergent periods.

**Objectives:**

This review aimed to explore disability considerations and accommodations included by African governments in their health systems’ responses during the COVID-19 pandemic.

**Method:**

A scoping review was carried out of peer-reviewed published articles on the Web of Science, Academic Search Premier, MEDLINE, Africa-Wide Info, and CINAHL databases. A desktop search of African government websites for COVID-19 country plans and reports was also conducted. Deductive thematic analysis of included texts was performed to identify disability inclusiveness in the health responses.

**Results:**

Ten peer-reviewed articles and three COVID-19 country plans or reports were included in the review. Data reflected a general finding that included countries that failed to effectively consider and include the healthcare needs of persons with disabilities during the pandemic.

**Conclusion:**

Poor inclusion of persons with disabilities was effected in healthcare systems’ responses during COVID-19 in Africa.

**Contribution:**

This article contributed insights about gaps in healthcare systems’ responses and highlighted development foci that could improve systems towards greater inclusivity of persons with disabilities’ health needs in low- and middle-income countries.

## Introduction

People with disability are often excluded from policy actions during emergency responses to crises situations (McKinney, McKinney & Swartz 2020; Ned et al. 2020; Toquero 2020). Prior to the coronavirus disease 2019 (COVID-19) pandemic, studies of disaster risk frameworks showed that people with disabilities are not explicitly considered in such frameworks, despite the adoption of the Sendai Framework Disaster Risk Reduction 2015–2030 that guides inclusive disaster risk responses (Bennett 2020). For example, a study of Indonesian disaster regulation laws found that people with disabilities were not mentioned in higher laws, and were often referred to as ‘vulnerable groups’ or mentioned with other vulnerable groups (Pertiwi, Llewellyn & Villeneuve 2020:3). With climate change, social instability, and new and emerging infectious diseases likely to cause more global health emergencies as observed during COVID-19, there is a need to continuously examine disaster responses to realise inclusion and rights of people with disabilities. Both the pandemic and its measures for containment, if not inclusive, pose threats to people with disabilities. Thus, more research is needed to explore the inclusiveness of health systems during the COVID-19 pandemic, more so in LMICs (low- and middle income countries) as 80% of the 16% global population with disabilities live in LMICs (World Health Organization [WHO] 2022).

The COVID-19 pandemic caused major disruptions in global health systems and challenged governments and health systems to make quick policy decisions to reduce the virus spread and curb morbidity and mortality associated with the virus (Haldane et al. 2021). Health systems had to ensure availability of hospital beds and staff to deal with the COVID-19 patient loads while preventing complete health system collapse (Haldane et al. 2021). For example, many countries around the world restricted the use of hospital services by cancelling and rescheduling elective and non-urgent procedures (Desborough et al. 2020; Kendzerska et al. 2021) as well as through diverting other health services and facilities to respond to the pandemic. School closures similarly impacted the health and function of people who received such health services through schools. People with disability were particularly affected by these changes in routine services because many people with disabilities have higher health needs including rehabilitation, medications, and other specialist services (Ned et al. 2020, 2021). In addition, some hospitals transitioned to remote services through telemedicine and closed certain wards and units to establish and allocate resources for COVID-19 patients (Desborough et al. 2020; Haldane et al. 2021). Although telemedicine plays a significant role in decreasing extra costs for some, such as travel costs, while also enhancing access for others, its limited availability within public health settings, poor connectivity, as well as an existing digital divide means that it may not be appropriate for a huge section of society in remote settings and for specific impairments. Triage systems were used to prioritise patients for healthcare through considering travel history, severity of COVID-19 symptoms, and patients’ risk profile (Kendzerska et al. 2021). Although these systems were effective for optimising human and material resources for COVID-19 patients’ care, certain groups of patients such as those who required chronic care were excluded (Kendzerska et al. 2021; Sabatello et al. 2020). People with disabilities constitute one group, which was vulnerable to such exclusionary COVID-19 policies. As such, some people with disabilities faced barriers while attempting to access vaccines including booking appointments, travelling to vaccination sites for vaccines and inaccessibility of such sites (Rotenberg & Nagesh 2021).

Sabatello et al. (2020) highlighted three thematic areas of exclusion that mostly affected people with disabilities during the pandemic, namely communication and medical information, reasonable accommodation, and rationing of medical goods and services. In the first place, studies show that many people with disability had challenges accessing health information from governments and health organisations, such as the WHO, because of the lack of accommodations like sign language interpreters and subtitles (Croft & Fraser 2022; Fernández-Díaz, Iglesias-Sánchez & Jambrino-Maldonado 2020). Secondly, many hospitals imposed restrictions that limited the number of visitors, preventing people with disabilities from receiving support from caregivers and/or personal assistants in the absence of reasonable accommodations of their visitor needs (Sabatello et al. 2020). Many hospitals constructed new buildings and/or rearranged existing structures to accommodate the high demand of COVID-19 patients and care. The emergency context under which alternative care structures were developed resulted in a disregard of physical accessibility needs although it was not necessarily regarded as a violation of anti-discrimination laws under emergency circumstances (Sabatello et al. 2020). Insufficient planning in relation to reasonable accommodations led to the exclusion of persons with hearing impairment when transparent face masks for lip-reading were not available during the pandemic (Sabatello et al. 2020).

Thirdly, aspect of exclusion was through rationing of health services and equipment. Triage policies of health systems in many countries specifically excluded people with certain disabilities; for example, one North American state originally excluded people with ‘severe or profound mental retardation’, moderate to severe dementia, and traumatic brain injury from ventilator treatment during the pandemic (Mello, Persad & White 2020:1). In addition, the reduction of health services to prioritise space and human resources for COVID-19 also severely impacted people with disabilities as they struggled to access routine health rehabilitation services (Agbelie 2023; Lund & Ayers 2022; Tetali et al. 2022).

Disability is not routinely considered when planning for health services (Hunt 2020; Ned et al. 2020). Sabatello et al. (2020) call for improved disability inclusive policies to ensure that people with disabilities who already experience discrimination in accessing services are not further excluded by COVID-19 health policies. Even health systems that responded relatively well to the COVID-19 pandemic had severe shortcomings in relation to disability inclusion. For example, an Australian study found that laws that addressed people who experience multiple exclusion were applicable to people with disabilities, but not specifically for people with disabilities, and thus failed to address the specific needs of people with disabilities (Colon-Cabrera et al. 2021). These laws failed to address the lack of access to healthcare, employment and social care, and unfair discrimination in medical rationing (Colon-Cabrera et al. 2021). A South American analysis found good practices in the articulation of disability specific policies; however, the implementation of these practices was not always adequate (Sakellariou, Malfitano & Rotarou 2020). Challenges identified with the implementation of such policies include discrepancies between national and local government policies, and the benefits not reaching all people with disabilities because of structural barriers (Sakellariou et al. 2020). These findings highlight the importance of not only articulating disability inclusive policies but also ensuring implementation and equity within communities of people with disabilities (Sakellariou et al. 2020). A study conducted in four West African countries also found that the governments’ efforts did not adequately address the needs of people with disabilities in the pandemic even though some efforts from these governments were recorded (Aboagye et al. 2022). Similar to the South American study, people with disabilities experienced structural barriers such as being unregistered with government disability agencies (Aboagye et al. 2022).

Disability inclusive policies are even more crucial for people with disabilities in LMIC countries, particularly in Africa, where health systems are strained and under-resourced. So far research that examines African governments’ health system responses in a holistic way is scarce. Aside from the study performed by Aboagye and colleagues of four West African countries (2022), there are no published holistic examinations of broader health system and policy responses from this context. The majority of available studies focus on specific aspects and experiences of people with disabilities, such as barriers to accessing health services, while there is a paucity of research about underlying notions in policies that create and reinforce these barriers. Against this backdrop, the authors conducted a scoping review on the overview of the health system government responses in relation to disability considerations in Africa during the COVID-19 pandemic from March 2020 to December 2022. The specific question asked was ‘How disability inclusive were the health systems in Africa in their COVID-19 pandemic responses from March 2020 to December 2022?’. Although the state of national disaster was called off in March 2022, the authors envisioned that there may still be literature published until December of the same year, hence they extended the timeline.

## Methodology

**FIGURE 1:**
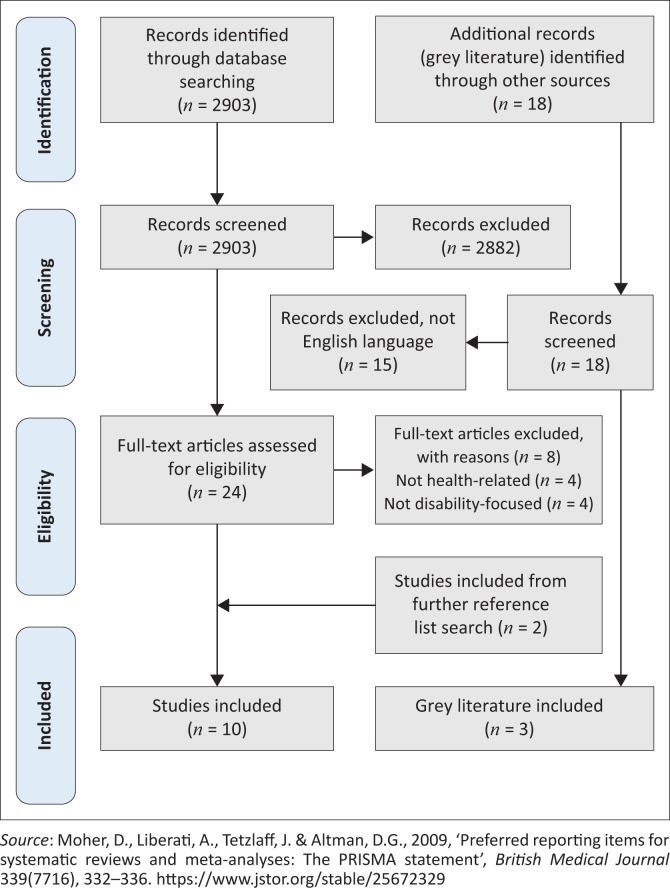
PRISMA Flow Diagram of the scoping review process.

The authors conducted a scoping review of peer reviewed literature and grey literature on health responses to COVID-19 across all African countries, informed by Arksey and O’Malley’s methodological framework (2005) and Levac, Colquhoun and O’Brien (2010), and is reported according to the Preferred Reporting Items for Systematic Reviews and Meta-Analyses extension for Scoping Reviews (PRISMA-ScR) (Moher et al., 2009) (see Figure 1). A scoping review is better able to provide a sense of breadth and depth of a body of research or field and was thus found appropriate to answer the question in this study. The objectives of the scoping review were four-fold:

- To summarise and map available peer-reviewed literature from March 2020 to December 2022 on the response of health systems in Africa
- To synthesise and describe the reported responses across countries using the UN and WHO action guidelines for governments
- To identify gaps within the health system responses in Africa
- To make recommendations for future pandemic responses in Africa.

### Searching peer reviewed literature

A systematic search was conducted on 10 databases (PubMed, Scopus, Web of Science (Social Sciences Citation Index), Africa-Wide Information, PROSPERO, ERIC, MEDLINE, CINAHL, PsycArticles, and Academic Search Premier). The peer reviewed literature search was run in English and the parameters were March 2020 to December 2022. Relevant Medical Subject Headings (MeSH) terms and keywords were developed and identified in collaboration with a librarian based on a preliminary examination of the literature and previous reviews conducted on related topics. Terms were grouped into four themes using PECO, namely population (all people with disabilities), Exposure (COVID-19 pandemic including COVID-19 vaccines), Context (Africa), Outcomes (health system responses, disability considerations, disability accommodations and disability inclusive responses). The population and location terms were based on recent Campbell Collaboration search strategies for evidence gap maps on disability inclusive development in LMICs. The first run of searches was performed in June 2022 and a re-run was completed in January 2023. The full search strategy is available from authors upon request. Studies were included if they described any type of healthcare government responses or measures to COVID-19 for persons with disabilities, for example, accessible public health information and communication, protective measures against COVID-19, accessibility to services, reasonable accommodations, allocation of scarce medical resources, and mental health interventions inclusive of persons with disabilities. Studies that described experiences of people with disabilities in relation to these government health responses were also included. The search included all types of studies and articles addressing any group of, or all types of disabilities.

### Searching for grey literature

Evidence in the form of grey literature was included through a review of country pandemic plans and reports. Government websites across all African countries were searched to identify and download existing country COVID-19 plans and reports. The grey literature search was also run in English. Search parameters included technical research reports and country plans that described any type of government healthcare responses or other measures regarding COVID-19 for persons with disabilities (e.g., accessible public health information and communication) that were published or promulgated between March 2020 and December 2022 in the different African countries. This included any considerations or accommodation in relation to healthcare targeted at people with disabilities by governments across all African countries. Coronavirus disease 2019 legislation, policy documents, and reports from the non-governmental sector (NGOs) were excluded but addressed in a separate study.

Studies pertaining to other systems or ministries than health were also excluded from the review. Where no full text was available of original research, reviews, meta-analyses, and conference abstracts or posters, studies were excluded from this review, as well as studies published before or after March 2020 and December 2022. Studies published in languages other than English were excluded as the review team had limited ability to undertake analysis in other languages.

### Study screening, selection, and extraction

All search results were uploaded onto Rayyan.ai, a software package used to organise and conduct reviews. Duplicates were removed before first screening of titles and abstracts, which was performed on Rayyan.ai by three reviewers (L.M., Y.N., L.N.) independently and conflicts were resolved by the third reviewer (L.N.). Excel was further used for bibliographic management, screening, coding, and data synthesis. The reasons for exclusion of articles were recorded. This was followed by a full text screening of all included titles, which was also performed by all three reviewers. Further articles were excluded after full text screening and all reviewers agreed. Reference lists of articles identified by the search were also examined for eligible publications.

A data extraction sheet was developed to record information from reviewed articles, including year of publication, type of literature, target population, scope, and location. The authors further mined for information relevant to the key questions of the review as well as the predetermined themes. Three reviewers performed data extraction. A short summary of each article was produced containing information about the responses, which are reported on.

### Analysis

A deductive thematic analysis was performed to provide an overview of different types of healthcare responses, and of how persons with disabilities were prioritised or considered in responses. A thematic framework (see Table 1) was derived from the WHO brief on disability considerations during the COVID-19 outbreak (2020) as well as from the UN brief on disability inclusive responses to COVID-19 (2020), and the documents were analysed according to nine themes. Descriptive analysis explored common themes across the different bodies of evidence to determine similarities, differences, and gaps in the inclusion of persons with disabilities in actions across different healthcare contexts and governments, as well as perceptions of disability inclusive responses during the COVID-19 pandemic across countries.

**TABLE 1:** Thematic framework: Key elements for disability-inclusive response to COVID-19.

Methodological theme	Key element for disability-inclusive response to COVID-19
Ensure accessible public health information	Provision of all information in accessible formats, including sign language translation, Braille script, captioning and easy read. Ensuring that information is up to date.
Implement protective measures against COVID-19	Access to appropriate WASH facilities. Providing protective measure to those supporting PWDs. The distribution of personal protective equipment to persons with disabilities needs to be tailored to their impairment.
Ensure accessibility to services	Facilitating access to health services especially essential services. Removal of financial barriers to care. Measures taken to ensure equitable access to healthcare, including measures addressing disability-based discrimination.
Ensure non-discrimination in the allocation of scarce medical resources	Mitigate the risk of discriminatory decisions in resource allocation that put people with disabilities at a high level of disadvantage.
Make mental health interventions inclusive of persons with disabilities	Mental health and psychosocial support need to be accessible and not discriminate against persons with disabilities.
Ensure the continuity of support services	Develop and implement service continuity plans, particularly for people with disabilities with high support needs, as well as measures to reduce potential exposure to COVID-19 during the provision of services.
Reasonable accommodations for people with disabilities	Adjustments to public health measures to accommodate the needs of people with disabilities, including flexibility in restrictions on movement in public spaces.
Consideration of the needs of people with disabilities who face multiple exclusions	Measures taken to protect people with disabilities who are at an increased risk of social exclusion and poverty, such as women, children, homeless people and prisoners.
Protection of people living in residential settings	Measures taken to ensure people living in residential care are protected from infection.

*Source:* Adapted from World Health Organization, 2020, *Disability considerations during the COVID-19 outbreak*, viewed 23 January 2023, from https://iris.who.int/bitstream/handle/10665/332015/WHO-2019-nCov-Disability-2020.1-eng.pdf?sequence=1; United Nations, 2020, *Policy brief: A disability-inclusive response to COVID-19*, United Nations, from https://www.un.org/sites/un2.un.org/files/2020/05/sg_policy_brief_on_persons_with_disabilities_final.pdf; Sakellariou, D., Malfitano, A.P.S. & Rotarou, E.S., 2020, ‘Disability inclusiveness of government responses to COVID-19 in South America: A framework analysis study’, *International Journal for Equity in Health 19*, 1–10. https://doi.org/10.1186/s12939-020-01244-xorg/10.1186/s12939-020-01244-x

COVID-19, coronavirus disease 2019; PWD, people with disabilities; WASH, Water, Sanitation, and Hygiene.

### Ethical considerations

Primary data collection and informed consent procedures were not applicable in this review, but ethics approval for the broad study was obtained from Stellenbosch University Social, Behavioural and Education Research Ethics Committee (REC: SBE), project number 15244. Institutional documents that are part of grey literature are publicly available.

## Findings

The results of the search and screening processes for both bodies of literature are presented in the PRISMA flow chart (see Figure 1). The first search produced 2903 peer reviewed articles and 18 country reports for screening. After titles and abstracts were screened, 2882 articles as well as 15 non-English language country reports were excluded. The full text of the remaining 24 articles was assessed, and 16 of these were excluded because of not being health-related or not having a disability focus. The reference lists of full text articles were searched for further eligible literature, and two (*n* = 2) articles were included from this search. Ten peer-reviewed articles and three country reports (grey literature) were, thus, included in this review.

Peer-reviewed literature reported studies performed in 10 African countries, with South Africa, Ghana, Uganda, Cape Verde, Kenya, and Nigeria each represented in two or more studies. Three studies reported qualitative research, two used mixed methods, and another two reported literature reviews. The remaining studies comprised two opinion articles, a policy analysis, and one article that reported following a narrative approach to identifying country COVID-19 responses. No quantitative studies were found and included in the review. The included grey literature reported on both qualitative and quantitative data and comprised reports from three African countries. These were all from southern African countries, as other country reports identified were not accessible in English.

Findings were primarily reported in descriptive form and reflected analyses of how disability-inclusive African government responses were to the COVID-19 pandemic. It was interesting to see that the included articles were pointing out gaps and challenges and offered suggestions and recommendations, rather than documenting what the governments are doing in response to shortfalls. Table 2 presents the government responses in each country.

**TABLE 2:** African countries’ healthcare system responses to COVID-19.

Actions	Uganda	Zambia	Kenya	South Africa	Nigeria	Ethiopia	Tanzania	Ghana	Senegal	Cape Verde
Ensure accessible public health information	Mbazzi et al. 2022: Government provided information in English and Luganda (local language). Information was received through TV, radio and phone messages but not in accessible formats and families had to simplify and/or translate for the relative with communication difficulties	Wickenden et al. 2022:- Zambia provided sign language for all television updates from the Ministry of Health and COVID-19 programmes (but no reference provided for this) *Country report:* Government planned key activities for Communication and Community engagement: To conduct rapid behaviour assessment to understand key target audience, perceptions, concerns, influencers and preferred communication channels including the establishment of a specific coordination outreach platform for at risk groups who included PWDs To develop public outreach and awareness campaigns through sensitisation and adapted messages related to COVID-19 outbreak with a focus on prevention measures, including sign language, targeting LNOB groups (Elderly, Women, Youth, People with Disabilities, LGBT, immune-deficient, HIV positive and TB, Sex Workers, Inmates, Shanty Compound Dwellers, Homeless People/street children, People living in remote rural areas, migrants, refugees, health workers and other vulnerable groups)	-	Wickenden et al. 2022: Regulations issued by the Minister of Communications and Digital Technologies classified digital and broadcast technologies as an essential service These same regulations included sign language as one of the local languages mandatory in communicating COVID-19 messages	Lugo-Agudelo et al. 2022: Authors reference a resource where people with visual impairment are highlighted in ‘Measures for people with visual impairment in the COVID-19 pandemic’ in the domain of ‘prevention of contagion’ and ‘special information for visual impairment’ Aboagye et al. 2022: Concurrent sign language interpretation provided through TV programmes Sign language interpretation through a dedicated online channel	Lugo-Agudelo et al. 2022: Authors reference a government resource (Coronavirus and Deafness in Ethiopia) that cites ‘Measures for people with hearing impairment in the COVID-19’ under ‘Healthcare service’	-	Swanwick et al. 2020: Sign language interpretation of COVID-19 information on all national broadcasts. Aboagye et al. 2022: Information directly related to people with disabilities provided sign-language interpretation, and through a dedicated online channel. Concurrent sign language interpretation provided through TV programmes *Country report:* Report stated Interpersonal Communication (IPC)/Group Education sessions with people living with disability and HIV, and in all 47 prisons – no specific detail regarding the accessibility of the means of communication	Aboagye et al. 2022: Concurrent sign language interpretation provided on TV programmes	Aboagye et al. 2022: Daily press conferences and TV programmes on COVID-19 in sign language
Implement protective measures against COVID-19	-	*Country report*: Government set a specific objective to protect all citizens including the most vulnerable in society (such as the elderly, pregnant women, adolescents, people with disability, migrants, refugees and people with existing medical conditions) and identified PWDs as ‘at risk population’	-	Lugo-Agudelo et al. 2022: SA government issued safety precautions related to basic sighted guide skills regarding COVID-19 in a resource pertaining ‘Measures for people with visual impairment in the COVID-19 pandemic’ within the domains of ‘prevention of contagion’ and ‘special assistance’ Wickenden et al. 2022: Department of Basic Education provided guidelines for learners with different impairments for prevention of the spread of the virus during phased return to school processes	Aboagye et al. 2022: Government planned for providing of Personal Protective Equipments (PPEs) to persons with disabilities	-	Mohamed, Wamera & Malima 2022: Government provided face masks and sanitisers to PWDs but did not reach everyone	Aboagye et al. 2022: Planned for providing of PPEs to persons with disabilities	-	Aboagye et al. 2022: Planned for provision of PPEs to persons with disabilities
Ensure accessibility to services	-	Lugo-Agudelo et al. 2022: Zambian government issued precautions for COVID-19 related to wheelchair users and users of assistive technology Authors reference the resource as citing ‘Measures for people with physical impairment in the COVID-19 pandemic’ under ‘Assistive devices’ Reference made to research exploring the impact of the COVID-19 outbreak on vulnerable groups, including persons with disabilities, to ensure no one is left behind *Country report:* Mentioned provision of livelihood means to people with disabilities as a vulnerable group	Ressa 2021: - A reference to the National Council for Persons with Disability reports that the government implemented the Persons with Severe Disability Cash Transfer program from May to June 2020, paying each beneficiary $40/month	McKinney, McKinney & Swartz 2021: Evidence of inclusion of disability organisations in working groups and committees tasked with responding to the pandemic. Some groups (Presidential Working Group on Disability, SADA, and DPSA) had opportunities to meet with representatives of the State. BlindSA presented recommendations for mainstreaming disability in pandemic responses to the government. No further detail is given regarding responses from government and implementation of the recommendations.Wickenden et al. 2022: Regulations from Minister of Social Development stipulated a top-up of disability grants with R350 monthly (initially for 6 months), and modified requirements for medical reports Regulations from Minister for Cooperative Governance and Traditional Affairs stated that lockdown regulations permitted the continuation of private domestic live-in staff and staff providing care to sick, mentally ill, elderly, people with disabilities and children – recognising such staff as essential workers Regulations from Minister of Communications and Digital Technologies stipulated home delivery of medical products by pharmacies should be permitted Home delivery of medical products by pharmacies was permitted during lockdown Hlongwane et al. 2022: Disability grant increased with R350/month for an initial 6 months	Lugo-Agudelo et al. 2022: Authors refer to a government resource (Federal Ministry of Health, Nigeria Centre for Disease Control, Advisory for Vulnerable Groups – The Elderly and Those with Pre-existing Medical Conditions) which cites ‘Measures for people with impaired cardiopulmonary function during the COVID-19 pandemic’ under ‘Prevention’ *Country report:* Persons with disabilities mentioned as a vulnerable group under thematic area: Security, Logistics and Mass Care Number of households reached with social protection and humanitarian interventions (including palliatives) during the pandemic period was used as indicator Measure describes the social and humanitarian interventions provided to vulnerable groups and communities affected by the COVID-19 measures imposed. It is disaggregated by programme/intervention and vulnerability of persons of concern (Vulnerable Households; Persons with Disabilities; The Unemployed, The Elderly, IDPs and others)	-	-	Aboagye et al. 2022: Telemedicine available for people with COVID-19 related symptoms – unclear if there was specific focus on PWDs *Country report:* Federation of Disability was allocated a budget and the following stated as part of responses in education sector: The Risk communication team in collaboration with the federation of disability will engage people living with disability at the national, regional and district levels.	-	-
Ensure non-discrimination in the allocation of scarce medical resources	-	Kapiriri et al. 2022:- Zambia included people with disabilities as a vulnerable group in their priority setting plans. No further information about this was included	-	-	-	-	-	-	-	-
Make mental health interventions inclusive of persons with disabilities	-	-	-	Wickenden et al. 2022: Minister of Social Development issued regulations stipulating that caregivers should be available to provide care in residential facilities and for required home-based care services during the lockdown period – including psychosocial support services, which must be provided to all those infected with or affected by COVID-19 The same minister stipulated that people with disabilities requiring psychosocial interventions should have access to all prescribed medications and counselling as a minimum requirement for crisis interventions	-	-	-	-	-	Aboagye et al. 2022:- Mentions provision of counselling services and mental wellness checks for persons with disabilities
Ensure the continuity of support services	-	-	-	Wickenden et al. 2022: Minister of Social Development issued regulations stipulating personal assistance must be available to people with disabilities at all service points, hospitals, screening, testing facilities, supermarkets and any other appropriate available facilities The same minister agreed that, where deemed necessary, people with disabilities may be provided with regular care-giving services at their places of residence	Aboagye et al. 2022: Caregivers of persons with disabilities were permitted to go to work, even in areas that were under lockdown	-	-	Aboagye et al. 2022: Caregivers of people with disabilities were permitted to go to work, even in areas that were under lockdown	Aboagye et al. 2022: Stipulated the formation of a community network to identify people with disabilities in the community, monitor their well- being, and offer support Caregivers of people with disabilities were permitted to go to work, even in areas that were under lockdown	Aboagye et al. 2022: Caregivers of people with disabilities were permitted to go to work, even in areas that were under lockdown
Reasonable accommodations for people with disabilities	-	-	-	-	-	-	-	-	-	-
Consideration of the needs of people with disabilities who face multiple exclusions	-	-	-	Wickenden et al. 2022: Minister of Social Development stipulated that caregivers had to be available to provide care in residential facilities and for required home-based care services during the lockdown period, including psychosocial support services, which had to be provided to all those infected with or affected by COVID-19. The same minister stated that people with disabilities requiring psychosocial interventions had to have access to all prescribed medications and counselling as a minimum requirement for crisis interventions. Minister of Justice and Correctional Services stipulated that support should be available to persons with disabilities in all courts, court precincts and justice service points	-	-	-	-	-	-
Protection of people living in residential settings	-	-	-	Wickenden et al. 2022: Minister of Social Development agreed that when a person with disability was to be released from department of social development (DSD)-operated facilities, such as high care facilities in old age person (OAP) homes, as well as drug and alcohol rehabilitation centres, a social worker should have been satisfied with regard to the state of readiness of the place that will accommodate that person	-	-	-	-	-	-

COVID-19, coronavirus disease 2019.

The aim of this review was to examine how inclusive government health systems’ responses were of persons with disabilities during COVID-19 in African countries. All but two of the included countries reported measures to ensure that public health information related to the pandemic was accessible to persons with disabilities. Most notably, sign language interpretation was used to make visual broadcasting about COVID-19-related information and precautions accessible to people with disabilities in six countries (Aboagye et al. 2022; Swanwick et al. 2020; Wickenden et al. 2022), and in South Africa, services using digital and broadcast technologies were classified as essential services (Wickenden et al. 2022). A Nigerian resource alluded to measures for people with visual impairment in the domain of preventing contagion with the virus, but with no further information about these measures reported (Lugo-Agudelo et al. 2022). The Zambian government included people with disabilities as one of the at-risk groups in their planning of key activities for communication and community engagement during the pandemic (Republic of Zambia 2020).

Six countries intensified protective measures against COVID-19 through, specifically, issuing regulations for the provision and utilisation of personal protective equipment (PPE). Nigeria, Tanzania, Ghana, and Cape Verde included the provision of PPE to people with disabilities in their documented responses to the pandemic (Aboagye et al. 2022; Mohamed et al. 2022). Researchers reported, however, that not all Tanzanians with disabilities received face masks and sanitiser (Mohamed et al. 2022). While not directly from the Ministry of Health, the South African Department of Basic Education published health-related guidelines for the prevention of the spread of the virus during the phased return to school for learners with different types of impairments (Wickenden et al. 2022). Only South Africa appeared to have issued safety precautions related to persons with specific impairments, namely visual impairments (Lugo-Agudelo et al. 2022).

Although access to healthcare is a human right, barriers to equitable healthcare remain one of the major challenges to people with disabilities (Badu, Agyei-Baffour & Peprah Opoku 2016). The COVID-19 pandemic intensified the nature of challenges that people with disabilities experienced in accessing healthcare services. Five countries in this review responded in varying degrees to this principle, while other countries did not reflect any specific responses to ensuring healthcare access for persons with disabilities. Zambia and Nigeria grouped citizens with disabilities together with other vulnerable groups, giving them access to social and humanitarian means in these countries (Presidential Task Force 2021; Republic of Zambia 2020). No further information was available, however, about the operationalisation priority measures for people with disabilities.

From this review, most documented evidence of measures to ensure access to healthcare services for persons with disabilities during the pandemic came from South Africa. Here, regulations issued by the Minister of Social Development that pertained to persons with disabilities may have indirectly promoted their access to healthcare services. The ministry, for example, extended the validity of medical reports, needed for the renewal of disability grants, from 3 to 6 months during the pandemic, and permitted live-in staff who provided care to persons with disabilities to remain in service during lockdown periods as providers of essential services (Wickenden et al. 2022). Temporary disability grants, therefore, that would have lapsed and needed to be reapplied for during lockdown periods, were extended until December 2020 without interruption of payments (Wickenden et al. 2022).

South Africa and Kenya increased social grants to persons with disabilities during the pandemic. In Kenya, the government implemented the Persons with Severe Disability Cash Transfer Program for 2 months, with each beneficiary receiving $40/month (Ressa 2021). Hlongwane et al. (2022) reported that the disability grant paid to South Africans with disabilities was topped up by the government with R350 per month (22 US dollars at the time) initially for a period of 6 months. Another South African social grant, a Caregivers Allowance, was made available from June 2020 to October 2020 to caregivers of children, but not to those who cared for persons with disabilities (Wickenden et al. 2022). Reviewed documents did not report regulations pertaining new disability grant applicants and how such applications were processed during lockdown restriction periods and thereafter.

Furthermore, the South African Minister of Communications and Digital Technologies classified services related to digital and broadcasting technologies as essential services in combatting the spread of COVID-19 (Wickenden et al. 2022). The ministry directed that electronic communications and broadcast licensees support the health sector by, for example, zero-rating Department of Health COVID-19 sites as well as calls to the department’s national helpline (Wickenden et al. 2022). The same ministry enabled pharmacies to deliver medical products at home during various levels of lockdown, which included but were not specific to persons with disabilities (Wickenden et al. 2022).

Some evidence suggested that the South African Government engaged disability organisations in working groups and committees who were tasked with developing healthcare responses to the pandemic. (McKinney et al. 2021). No evidence was available about the implementation of recommendations from disability stakeholders.

The pandemic brought a global sense of panic and heightened anxiety and even in the early days of the crisis the emergence of mental health concerns was noticed by health authorities. Only two countries in this review appeared to have included persons with disabilities to some degree in their healthcare responses in mental health interventions during the pandemic. The South African Minister of Social Development instructed that support from caregivers in residential facilities and/or homes during lockdown periods should include psychosocial assistance to those infected with or affected by COVID-19 during lockdown periods (Wickenden et al. 2022). The same ministry also stipulated that persons with disabilities who required psychosocial intervention should have access to prescribed medication and counselling as a minimum requirement during the crisis intervention period (Wickenden et al. 2022). It is noticed that these directives were not set by the government department who is primarily responsible for mental healthcare services, and as such, these were issued to the Department of Health rather than to service providers directly. Cape Verde’s country report stipulated the provision of counselling services to persons with disabilities as a response during the pandemic, including assessing this group’s ‘mental wellness’ (Aboagye et al. 2022:7).

Five reviewed countries showed evidence of efforts to ensure continuity of support services to persons with disabilities during the pandemic. In these countries, caregivers were allowed to continue with service provision to people with disabilities under lockdown restrictions (Aboagye et al. 2022; Wickenden et al. 2022). Additionally, in Senegal, networks were formed in communities to identify persons with disabilities, monitor their well-being, and offer support (Aboagye et al. 2022), and South Africa issued regulations about access to personal assistance for persons with disabilities at various facilities, such as service points and supermarkets (Wickenden et al. 2022).

None of the included countries reported measures to reasonably accommodate persons with disabilities in relation to public health services and restrictions on movement in public spaces. Furthermore, documents included did not address how countries protected people with disabilities from discriminatory decisions around medical resource allocation, apart from Zambia’s inclusion of persons with disabilities in vulnerable groups while setting priority plans during the pandemic (Kapiriri et al. 2022). No further information was found in the Zambian document, however, of how this inclusion realised for persons with disabilities. South Africa was the only country that mentioned measures to protect people with disabilities from increased social exclusion through issuing regulations for continued availability of caregivers in residential facilities and home-based environments during lockdown restrictions (Wickenden et al. 2022). Continued psychosocial support services and access to prescribed medications and counselling were also mentioned in regulations to mitigate social exclusion risk for this group. Unrelated to healthcare services, the South African Minister of Justice and Correctional Services issued regulations that permitted support persons to be available in courts, court precincts and justice service points to assist persons with disabilities (Wickenden et al. 2022). In addition, this ministry stated that inmates (including people with disabilities) could be referred to external health facilities only if medical emergencies occurred, circumstances under which access to healthcare may have been denied to inmates with long-term physical and/or mental health disabilities (Wickenden et al. 2022).

Only South African evidence included the protection of people in residential settings in the documents reviewed. The Minister of Social Development stated that a satisfactory assessment from a social worker should be a prerequisite for releasing persons with disabilities from Department of Social Development-operated facilities into different places of accommodation (Wickenden et al. 2022). This review did not find evidence of policy considerations or implementation pertaining to persons with disabilities’ access to healthcare systems and services in remote areas during the pandemic.

## Discussion

The health and lives of persons with disabilities have been disproportionately affected by the outbreak of the novel coronavirus 2019 and the ensuant pandemic (United Nations 2020). Adversity created by this global crisis had even more severe marginalisation and disadvantageous effects for people with disabilities from low- and middle-income countries (McKinney et al. 2021). This review considered how countries on the African continent included persons with disabilities in the responses of their healthcare systems during the pandemic.

The most consistent evidence found in this review was in the sampled countries’ promotion of access to public health information through the provision of sign language interpretation during visual broadcasts of COVID-19-related information and precautions. Although not all countries reported such inclusion measures, the ones who did may have mitigated the isolating effect of the pandemic for sign language users through their attention to alternative and/or augmented means of communication. The availability of and existing infrastructure to support sign language interpretation may have been seamlessly applied for the use of pandemic information-sharing because of these services having been operational before the crisis. Promoting access through other means, however, that accommodated the entire range of communication impairments, was not stipulated and may have resulted in exclusion of persons with disabilities, and also where broadcasting and digital accessibility were limited or unavailable.

Persons with disabilities did not have equitable access to healthcare and health information prior to COVID-19 (McKinney et al. 2021). Evidence from both high income and low- and middle-income countries confirms that people with disabilities are disadvantaged compared with those without disabilities in accessing health services (Reem Mutwali & Ross 2018). In Africa, people with disabilities navigate challenges of transport and distance, cost, waiting times, and physical accessibility of facilities, among others, resulting in lower overall utilisation of health services (Reem Mutwali & Ross 2018). Several authors have reported on the exacerbated challenges faced by persons with disabilities during COVID-19 as a result of governments’ failure to consider disability inclusion in their disaster response and disaster management plans (Jesus et al. 2021; McKinney et al. 2020; Sakellariou et al. 2020). Overwhelmingly, the evidence from this review reflects that included countries proffered minimal or no policy guidelines or measures for including persons with disabilities in healthcare accessibility initiatives during the pandemic. Where people with disabilities were highlighted or mentioned, they were often grouped together with other minority communities and subject to broad policy principles rather than targeted measures aimed at addressing their specific accessibility needs.

None of the countries overtly addressed reasonable adjustments to health measures that would accommodate the needs of persons with disabilities during the pandemic, although some application of reasonable accommodation (RA) principles may have been included by planned actions, for example, by allowing carers of persons with disabilities to attend work under lockdown circumstances. Reasonable accommodation refers to measures aimed at preventing generic prohibitions during the pandemic from inadvertently excluding and/or disadvantaging people with disabilities (UN Office of the High Commissioner for Human Rights 2020) because of the presence of impairment. Sakellariou et al. (2020) reported examples of RA measures employed in Argentina and Peru during COVID-19, as allowing walks outside their homes for people with disabilities and their carers without requiring special permission to do so. In Peru, people with disabilities were also given priority access to humanitarian supplies and all other resources from the State at all levels of public administration (e.g. water and food) (Sakellariou et al. 2020). The absence of relevant reasonable accommodation measures found in this review would have compounded existing barriers to access and discriminatory practices. Regulations issued by the South African government reflected some attempts to promote healthcare access for persons with disabilities during COVID-19, but grassroot research reflected major challenges to accessing healthcare by this group during the pandemic (McKinney 2021). McKinney’s study found how access to therapy, assistive devices, and specialised care was limited or suspended under lockdown regulations, and how communication with healthcare staff was impacted when support persons for persons with hearing impairment were not allowed to accompany them into healthcare facilities (2021). Her study continues to elaborate on several experienced barriers to healthcare access by South Africans with disabilities, such as inaccessible transport to healthcare facilities, and inaccessible or unaccommodating vaccine procedures (McKinney 2021).

The omission or under serving of persons with disabilities in healthcare extended to their exclusion from targeted mental health interventions during the pandemic and in its wake. Mental health concerns during COVID-19 related not only to care and intervention for persons with existing mental health conditions but also to health system planning to handle a ‘second pandemic’ of mental health crises as a result of the COVID-19 pandemic (Choi et al. 2020:340; Kola et al. 2021).

Countries included in the review, furthermore, generally failed to plan for the protection of people with disabilities in residential settings, and the healthcare needs of persons with disabilities living in rural areas. Rurality, in African countries that form part of the Global South, is known to create an intersection with disability that intensifies the disadvantages and exclusion of this group in healthcare systems (Ned et al. 2020). Overall, evidence from this review confirms that sampled African states failed to effectively include the needs of persons with disabilities in health system responses to the emergency of COVID-19. Where inclusions were specified, the implementation of these fell short and left persons with disabilities more exposed, vulnerable, and excluded from healthcare than non-disabled counterparts.

### Limitations of this review

Grey literature from African countries, that may have been relevant to this scoping review, was not included when only available in languages other than English. This may have limited the reflection of evidence to report on from non-English speaking African countries. The inclusion of grey literature, however, served only to expand on and supplement the peer review articles that were eligible for inclusion because of the limited number of articles found.

### Implications and recommendations

Sakellariou and colleagues make the important point that legislation and policy afford legal recognition and protection of the rights of people with disabilities, even if they do not always prevent discrimination against this group (2020). As such, the significance of inclusive laws, policy frameworks, and regulations to direct health system responses during emergencies should not be underestimated, and gaps in such laws need to be addressed. Comprehensively inclusive guidelines exist in the global arena to direct disability-related policy and implementation efforts, for example, the UN Policy Brief for a disability-inclusive response to COVID-19 (United Nations 2020), UNESCO’s reference report on policy responsiveness during COVID-19 and best practice guidelines (UNESCO 2021a; 2021b), and the United Nations’ guidance brief on protecting the rights of persons with disabilities during COVID-19 (UN Office of the High Commissioner for Human Rights 2020). The WHO (2022) suggests governance as one of the strategic entry points for disability inclusion in health systems. Further and renewed efforts to incorporate such guidelines by African Union and United Nations member states should be launched and sustained in preparation for greater and targeted policy inclusiveness of the health needs of persons with disabilities under non-emergent and emergent circumstances.

Regulations that are promulgated in the context of emergencies or disasters must be interpreted in order to be applied by those who implement them (Wickenden et al. 2022). It would follow that service providers’ knowledge and understanding of disability inclusion are crucial to the effective implementation of inclusive healthcare policies in practice.

This review has shown that, although governments in some instances heeded the principles of including and collaborating with organisations of people with disabilities to address and plan appropriately inclusive emergency responses, these occasions were insufficient and took place too late for effective implementation to run its course. Moreover, no evidence of outcomes or monitoring and evaluation reporting is available to ascertain what impact these deliberations had on healthcare inclusion, if any. Government and policymakers should therefore strengthen and incorporate collaborative relationships with organisations that represent people with disabilities to consistently influence planning forums at regional, local, and national government levels during and beyond emergency contexts.

Access to public health information should now move beyond governments’ standard response to communication impairments (such as sign language interpretation during visual broadcasts) and incorporate universal design principles to bolster sophisticated communication means in all ‘sensory channels’ (Wickenden et al. 2022:13). As such, attention should be paid to creating access to information for, for example, persons who do not read, or who require texts or messages to be shortened or simplified for comprehension.

## Conclusion

From this review of a sample of African countries’ health system responses to the inclusion needs of persons with disabilities during the COVID-19 pandemic, it is concluded that an overall poor inclusion response was stipulated. Countries appear to have been most responsive in terms of creating access to health information, and most neglectful in stipulating reasonable accommodation measures to equalise health system access and services to persons with disabilities. This review shows areas of concern and priorities where governments should develop health inclusive efforts in existing and future health systems that could address the needs of persons with disabilities as equal citizens during and beyond health emergencies.
